# Quality of Life and Side Effects Management in Cancer Treatment—A Cross Sectional Study

**DOI:** 10.3390/ijerph20031708

**Published:** 2023-01-17

**Authors:** Inés Llamas-Ramos, Jorge Juan Alvarado-Omenat, María Rodrigo-Reguilón, Rocío Llamas-Ramos

**Affiliations:** 1 Faculty of Nursing and Physiotherapy, Universidad de Salamanca, C/Donantes de Sangre s/n, 37007 Salamanca, Spain; 2 University Hospital of Salamanca, Paseo de San Vicente, 182, 37007 Salamanca, Spain; 3 Institute of Biomedical Research of Salamanca (IBSAL), 37007 Salamanca, Spain; 4 FisioSport, CB, C/12 de Octubre, 2, 37008 Salamanca, Spain; 5 Virgen de la Concha Hospital, Avda Requejo, 35, 49022 Zamora, Spain

**Keywords:** physical symptoms, psychological symptoms, quality of life, cancer

## Abstract

Cancer disease is a world problem which is increasing in its prevalence. Oncology patients have a multitude of symptoms derived from the treatments and from the disease itself that affect their quality of life to a greater or lesser extent. The aim of this study has been to discover the physical and psychological symptoms related to chemotherapy treatment in Spanish cancer patients in order to improve their quality of life. Symptoms from the previous week were taken into account and the Memorial Symptom Assessment Scale was used to measure the frequency, severity and associated distress of 32 symptoms. A total of 246 chemotherapy patients at the University Day Hospital in Salamanca completed the scale once while receiving chemotherapy treatment. A 95% confidence interval was considered. The most prevalent symptoms were a lack of energy (76.4%), anxiety (66.7%) and a dry mouth (60.6%). Lung cancer was the most prevalent cancer in men (26%) and breast cancer was the most prevalent cancer in women (72%). There is no consensus on which is the most prevalent symptom in this population and more studies will need to be carried out to determine the best treatment protocols. Symptom’s prevalence knowledge could improve the patients’ care to prevent or avoid complications and to improve the cancer patients’ quality of life.

## 1. Introduction

Cancer constitutes a huge burden for society [1,2], both in more developed countries and in less developed countries. Its prevalence is increasing due to population growth and aging, as well as a risk factors increase (tobacco or alcohol consumption, overweight/obesity, physical inactivity, or a change in reproductive factors associated with urbanization and economic development [3]).

In 2018, approximately 9.6 million tumor-related deaths were recorded, making cancer one of the most prevalent diseases globally if we consider the data provided by the World Health Organization (WHO). According to the latest information from the National Statistics Institute provided in December 2019 (about the year 2018), tumors were the second cause of death in Spain (26.4% of deaths) behind circulatory system diseases (28.3% of deaths). Lung and colon cancer tumors are the main causes responsible for these deaths. Regarding age, tumors were the main cause of death between one and 14 years (29.2% of the total) and between 40 and 79 years (43.8%). The most frequently diagnosed tumors in the world in 2018 were lung, breast, colon and rectum, prostate and stomach. The data published in the GLOBOCAN project accounted for 18.1 million new cases in the world in 2018 and an estimate by the Spanish Society of Oncology Medicine [4] is that the number of patients will rise to 29.5 million by 2040.

The new cancer cases diagnosed in Spain in 2020 will reach 277,394 patients, similar to 2019 (277,234), the most frequently diagnosed cancers being colon and rectum (44,231 new cases), prostate (35,126), breast (32,953), lung (29,638) and urinary bladder (22,350) [4].

These patients experience many symptoms from diagnosis to the end of treatment or end of life, which affect their quality of life. These symptoms not only depend on the disease itself but on the treatments received. Knowledge of these symptoms and their prevalence is essential to establish the basis of a good treatment to try to anticipate them and reduce their incidence [5].

Establishing the prevalence of symptoms in this population is just as important as choosing the appropriate instrument. There are multiple scales that measure the prevalence of a single symptom: the Brief Pain Inventory (BPI) [6], the Karnofsky Performance Status (KPS) [7], the Brief Fatigue Inventory (BFI) [8], the Perform Questionnaire (PQ) [9,10], the Hospital Anxiety and Depression Scale (HADS) [11], or the Constipation Assessment Scale (CAS) [12], among others. On the other hand, there are also instruments that assess more than one symptom: the Symptom Distress Scale (SDS) [13], the Edmonton Symptom Assessment Scale (ESAS) [14], the MD Anderson Symptom Inventory (MDASI) [15], the European Organization for Research and Treatment of Cancer Quality of Life Questionnaire-Core 30 (EORTC QLQ-C30) [16], the Rotterdam Symptom Checklist (RSCL) [17], or the Memorial Symptom Assessment Scale (MSAS) [18].

Some of these instruments show the responses using visual scales (the SDS, the ESAS, and the MDASI) and others present categorical descriptor responses (the RSCL, the EORTC QLQ-C30, and the MSAS).

Given the need to have specific instruments for a cancer patient’s assessment, the adaptation to Spanish of the Memorial Symptom Assessment Scale was made, in order to provide a categorical response tool in Spanish, to know the symptomatology and intensity of the symptoms in the three dimensions that evaluate the scale in cancer patients. To this end, the MSAS has been translated and validated into Spanish by Llamas-Ramos et al. [19]. This scale allowed us to collect the most information about the patient symptom experience due to chemotherapy treatments.

The objective of this study has been to establish the symptoms prevalence in oncology patients using the Spanish MSAS version. This knowledge may help to improve their quality of life during and after their oncology treatments.

## 2. Materials and Methods

### 2.1. Study Design

This study is a cross-sectional study. The patients were recruited from the University Hospital of Salamanca (Spain). The scale was filled in by the patients at the same hospital while they were receiving their chemotherapy treatment.

The study was approved by the Hospital Ethical Committee of Salamanca.

### 2.2. Sample

A convenient sample of patients who participated in the validation version of MSAS was analyzed. The study included patients aged from 18 to 85 years, diagnosed with cancer, and patients who attended to receive their chemotherapy treatment in the Day Hospital of the University Hospital of Salamanca. The sample procedure was consecutive once they met inclusion criteria. All participants were informed and signed an informed consent.

Patients who were receiving their first chemotherapy session, who were in the terminal phase of the disease, who presented cognitive impairment or any neuropsychological disability that prevented them from responding to the scale, who did not know how to read or write, or who suffered from severe hearing loss or blindness or who had not completed more than 13% of the MSAS (as the creator of the original version postulated), were excluded.

### 2.3. Data Collection Procedure

One trained researcher was the person who recruited the samples in person. She was available if patients had doubts and she collected the filled-in questionnaires before the chemotherapy session had finished. Another researcher was in charge of informing patients about the study, to avoid duplication in the participation, and if they agreed to participate, they received a code to guarantee the anonymity of the patients.

### 2.4. Questionnaire

The Memorial Symptom Assessment Scale (MSAS) was selected as the instrument to establish the prevalence of physical and psychological symptoms in cancer patients with active treatment.

The MSAS evaluates the frequency, severity and distress of 32 symptoms during the previous week and the severity and associated distress of another eight symptoms. This scale was translated into Spanish [19] and it is shown to be valid and reliable.

First, patients had to check the box “Did not have” if during the previous week they have not experienced the symptom, and if they have felt it, they must indicate the frequency with the options: 1 = “rarely”, 2 = “occasionally”, 3 = “frequently” and 4 = “almost constantly”; the severity of the symptom was classified with the options: 1 = “slight”, 2 = “moderate”, 3 = “severe” and 4 = “very severe”. In addition, the distress had the options: 0 = “not at all”, 1 = “a little bit”, 2 = “somewhat”, 3 = “quite a bit” and 4 = “very much”.

In the first two dimensions, frequency and severity, the score would be the value designated by the patient (from 1 to 4). In the distress dimension, which had five possible answers, the following equivalence was established: 0.8 = “not at all”; 1.6 = “a little bit”; 2.4 = “somewhat”; 3.2 = “quite a bit” and 4 = “very much”. The mean of the values obtained in each of the dimensions was the final score for a symptom. If any symptom had not been felt, it was assessed with a 0. The MSAS is composed of three subscales; the psychological subscale collects six psychological symptoms (feeling sad, feeling irritable, feeling nervous, worrying, difficulty concentrating and difficulty sleeping); the physical subscale is the principal of 12 physical symptoms (pain, lack of energy, feeling drowsy, nausea, dry mouth, lack of appetite, feeling bloated, constipation, changes in the way food tastes, dizziness, weight loss and vomiting); finally, the Global Distress Index is the principal index of the frequency of four psychological symptoms (feeling sad, feeling nervous, feeling irritable and worrying) and the distress of six physical symptoms (lack of energy, lack of appetite, pain, constipation, dry mouth, pain). Therefore, the total score was the average of the values of the 32 symptoms.

### 2.5. Statistical Analysis

A descriptive analysis of the demographic data of the sample was performed, as well as the symptoms’ prevalence. The mean, the standard deviation (SD), the 95% confidence interval (95% CI), the minimum and maximum for the quantitative variables and the counts and percentages for the qualitative variables were used.

## 3. Results

### 3.1. Sample

A total of 246 subjects participated in the study. Among the sample characteristics, it was found that 62.2% (n = 153) of the sample were women. The mean age of the patients was 59.98 years (SD = 11.696). A total of 66.3% (n = 163) were married, 41.9% (n = 103) had received primary education, and 11.4% (n = 28) were active workers (Table 1).

The types of cancer registered in our study were: colon, rectum, bladder, stomach, liver, kidney, pancreas, lung, breast, gynecological, prostate, testicle, hematological, head and neck, esophagus, melanoma and other more prevalent symptoms in each type of cancer.

The most frequent cancer types for men were: lung cancer in 28.0% (n = 26); followed by colon cancer in 22.6% (n = 21) and hematological cancer in 12.9% (n = 12). For women, breast cancer accounted for 47.1% of the sample (n = 72), followed by gynecological cancer with 14.4% (n = 22) and colon cancer with 7.2% (n = 11) (Table 1).

### 3.2. Description of the Symptoms of the Sample

For the sample recruited in this study, “lack of energy” turned out to be the most prevalent symptom in cancers of the colon, rectum, bladder, stomach, pancreas, lung, breast, hematological and in others different types of cancer that were less prevalent and collected in the study (Table 2).

In relation to physical and psychological symptoms, the number of symptoms experienced was 11.09 (SD 6.210). The minimum was 0 symptoms, and the maximum was 30 symptoms. On the other hand, the prevalence of symptoms ranged from 76.4% to 9.3%. In this sense, the highest prevalence was registered in the following symptoms: “lack of energy” (76.4%; n = 188), “worrying” (66.7%; n = 164) and “dry mouth” (60.6%; n = 149) and the lowest frequency was recorded for the symptoms: “dizziness” (15.0%; n = 37), “vomiting” (13.0%; n = 32) and “problems with urination” (9.3%; n = 23).

### 3.3. MSAS: Frequency

For this dimension, the frequencies that were recorded as “almost constantly” were for the symptoms “problems with sexual interest or activity” (38.4%; n = 38), “numbness/tingling in hands/feet” (23.4%, n = 29) and “difficulty sleeping” (20.5%; n = 26). The most prevalent symptoms experienced with a frequency of “rarely” were “dizziness” (27%; n = 10), “vomiting” (25%; n = 8) and “shortness of breath” (22%; n = 13). The symptoms in the last section do not refer to any frequency because they can be present or not (Table 3). 

### 3.4. MSAS: Severity

“Hair loss” (38.0%; n = 38), “problems with sexual interest or activity” (28.3%; n = 28) and “changes in the way food tasted” (18.0 %; n = 22) were the symptoms perceived with a very considerable severity. On the other hand, the symptoms that were more reflected as a slight severity were “difficulty concentrating” (50.7%; n = 37), “mouth sores” (47.4%; n = 27) and “cough” (45.9%; n = 28) (Table 3).

### 3.5. MSAS: Distress

Symptoms selected with considerable distress were: “hair loss” (27.0%; n = 27), “I do not look like myself” (20.5%; n = 8) and “problems with urination” (17.4%; n = 4), while the symptoms which showed “not at all” distress were “weight loss” (60%; n = 33), “feeling drowsy” (32.5%; n = 25) and “changes in skin” (29.4%; n = 20) (Table 3).

### 3.6. Total MSAS

In the TOTAL MSAS, the men who experienced the most symptoms were those with prostate cancer and for women, those with liver cancer.

In the psychological subscale, for men the cancer that caused the most symptoms was testicular cancer and for women it was liver cancer.

In the physical subscale, the cancer that caused the most symptoms for men was pancreatic cancer and for women, bladder cancer.

Finally, in the global distress index, the cancer for which men experienced the most symptoms was testicular cancer and in women, liver cancer.

## 4. Discussion

In Spain, the number of diagnosed cancers has continued to increase, establishing a probable relationship with the population increase (47,100,396 inhabitants in 2019), aging, exposure to risk factors (tobacco, alcohol, pollution, obesity, a sedentary lifestyle, etc.) and the increase in early detection protocols in colorectal, breast, cervix or prostate cancer. In recent years, the incidence of cancer in men appears to have stabilized and to have increased in women, probably due to the decrease in smoking in the former and an increase in the latter since 1970 [4]. However, in the present study the percentage of lung cancer in men continues to represent 26% while women register 6%.

In 2020, the most frequent cancers diagnosed in men in Spain were those of the prostate, colon and rectum, lung and urinary bladder, and breast and colon and rectum in women. These differences appear to be related to exposure to different risk factors (environmental and endogenous); even hormonal differences could be involved [4]. This agrees with our study: in Spain, the most prevalent cancers have been colon and hematological lung for men and breast, gynecological and colon for women.

Cancer is a major cause of morbidity with troubling symptoms that may be experienced due to the disease process itself, during treatment, or due to a lack of inadequate control of symptoms [20]. Its control is essential in cancer care to achieve an optimal quality of life [21]. Arseven et al. [22] have exposed the importance of the symptoms; their understanding and management at every stage of the disease process will mitigate its effect and improve the quality of life [22].

There is no agreement in the literature on which is the most prevalent symptom; for some authors, this symptom would be pain [23,24,25,26], for others, it was lack of energy or weakness, [27,28,29,30] dyspnea [31] or lack of appetite [32,33]. References have also been found in which psychological stress [34] registered as having the greatest importance.

Donnelly et al. [35] analyzed 37 symptoms (1000 advanced cancer patients), and concluded that the symptoms commonly associated with cancer were pain, fatigue and anorexia. In addition, weakness, anxiety, lack of energy, early satiety, constipation, and dyspnea were all present in 60–80% of patients with a moderate to severe score. For Potter et al. [20] (400 patients), the five most frequent symptoms in the palliative care unit, where 95% of the patients had a diagnosis of cancer, were pain (64%), anorexia (34%), constipation (32%), weakness (32%) and dyspnea (31%). In relation to the prevalence of symptoms in patients receiving chemotherapy [36] (4000 questionnaires completed by 462 patients), there were oral problems (21%), insomnia (19%), psychological disorders (15%), need for assistance in decision-making (14%), severe fatigue (8.2%) and severe loss of appetite (6.3%). Petterson et al. [37] also evaluated the prevalence, frequency, and symptoms severity as well as the distress they cause in colorectal cancer patients during initial chemotherapy treatment. Numbness or tingling in the hands or feet (64%), lack of energy (62%), drowsiness (49%), and nausea (45%) were the most prevalent symptoms. In gynecological cancers, the clinically important symptoms were dyspepsia, nausea and vomiting [35]. In another study (1640 patients from seven centers in five different countries), nausea was the most frequent symptom in gynecological and stomach cancers; nausea, constipation, and anorexia were prevalent in esophageal, stomach, and colorectal cancers. Weakness was the most frequent in hematological, colorectal, and esophageal cancers, while dyspnea was the most frequent in lung cancer [38].

In relation to gender, Donnelly et al. [35] showed that the most prevalent symptoms for men were dyspnea, hoarseness, hiccups and dysphagia; for women, they were anxiety, nausea, vomiting and early satiety. In another study, men reported dysphagia and insomnia while women reported nausea and vomiting [23]. On the other hand, male patients receiving chemotherapy reported significantly greater intensity of fatigue, dyspnea, loss of appetite, and drowsiness [36].

In this study, the age range was 18–85 years and due to the heterogeneous sample we could not establish comparisons between different age groups. Regarding age groups, the literature shows that younger patients report significantly greater intensity of pain and nausea [36]. Van Lancker et al. [39] reanalyzed this prevalence in people over 65 years of age. They identified 32 symptoms, of which the seven most prevalent were fatigue (77.8%), excretory symptoms (77.5%), urinary incontinence (71%), asthenia (66.7%), pain (66.3%), constipation (52.5%) and anxiety (50%). They found that the greatest limitation, which constitutes an exclusion criterion in their work, is the absence of a valid and reliable measuring instrument to identify these symptoms.

Therefore, symptoms knowledge is important because the appraisal stage and the psychological influences that these treatments have need to be considered in order to develop behavioral and individual interventions [40]. An important aspect to note is that the symptoms do not usually appear in isolation, but can develop at the same time, sometimes manifesting as up to eight symptoms at the same time [41,42]; some cancers even have their own set of symptoms according to some authors [43].

The importance of this article is in line with Kwekkeboom’s study [43] in which she highlighted the importance of knowledge of the patient’s symptoms and summarized them in three points: (1) symptoms knowledge prevents negative outcomes (e.g., depression), (2) it favors a more thorough analysis of the symptoms which may even help to prevent them and finally, (3) recognition of the symptoms favors a more appropriate management of them. However, no studies have established the grade to which these experienced symptoms affect the patients who are suffering them.

For that reason, this study has collected the most prevalent, severe and distress-inducing symptoms in order to prevent or avoid them as much as possible to improve the quality of life of these patients during their treatment and after.

### Limitations

The main limitation of the study was that all patients were evaluated by being asked about the week prior to the chemotherapy session; they could suffer different symptoms in different weeks of the disease. Chemotherapy cycles administered and their medication were not taken into account, and it could be a bias which interfered with the present results. Furthermore, comparing different age groups as well as differences between gender or cancer type should be considered in the future. 

## 5. Conclusions

Physical and psychological symptoms generated by cancer treatments affect quality of life. There is no consensus on which is the most prevalent symptom in this population. Although lack of energy, anxiety and a dry mouth are the most prevalent symptoms experienced by our patients, more studies in this line of work are recommended to control and relieve them in cancer patients in order to improve their quality of life.

## Figures and Tables

**Table 1:** Socio-demographic data of the complete sample and by sex.

Variable (n = 246)		Total (n = 246)	Male (n = 93)	Female (n = 153)
Age (Years) A		59.98 (11.696)	62.80 (9.497)	58.27 (12.572)
Marital Status ^B^				
	Single	35 (14.2)	9 (9.7)	26 (17.0)
	Married	163 (66.3)	74 (79.6)	89 (58.2)
	Divorced	17 (6.9)	6 (6.5)	11 (7.2)
	Widowed	31 (12.6)	4 (4.3)	27 (17.6)
Study Level ^B^				
	Primary	103 (41.9)	41 (44.1)	62 (40.5)
	Secondary	35 (14.2)	18 (19.4)	17 (11.1)
	High School	40 (16.3)	16 (17.2)	24 (15.7)
	University	68 (27.6)	18 (19.4)	50 (32.7)
Occupation ^B^				
	Active	28 (11.4)	9 (9.7)	19 (12.4)
	Temporary Disability	58 (23.6)	19 (20.4)	39 (25.5)
	Housewife	50 (20.3)		50 (32.7)
	Unemployed	13 (5.3)	5 (5.4)	8 (5.2)
	Retired	97 (39.4)	60 (64.5)	37 (24.2)
Type of Cancer ^B^				
	Colon	32 (13.0)	21 (22.6)	11 (7.2)
	Rectum	7 (2.8)	5 (5.4)	2 (1.3)
	Bladder	4 (1.6)	3 (3.2)	1 (0.7)
	Stomach	5 (2.0)	1 (1.1)	4 (2.6)
	Liver	4 (1.6)	2 (2.2)	2 (1.3)
	Kidney	5 (2.0)	4 (4.3)	1 (0.7)
	Pancreas	8 (3.3)	2 (2.2)	6 (3.9)
	Lung	32 (13.0)	26 (28.0)	6 (3.9)
	Breast	73 (29.7)	1 (1.1)	72 (47.1)
	Gynecological	22 (8.9)		22 (14.4)
	Prostate	5 (2.0)	5 (5.4)	
	Testicle	2 (0.8)	2 (2.2)	
	Hematological	22 (8.9)	12 (12.9)	10 (6.5)
	Head and neck	10 (4.1)	4 (4.3)	6 (3.9)
	Esophagus	2 (0.8)	1 (1.1)	1 (0.7)
	Melanoma	2 (0.8)	2 (2.2)	
	Others	11 (4.5)	2 (2.2)	9 (5.9)

^A^ Mean (Standard Deviation); ^B^ Number (percentage).

**Table 2:** Most prevalent symptom in each type of cancer.

Cancer Type	Most Prevalent Symptom	Median	Standard Deviation
Colon	Lack of energy	1.41	1.010
Rectum	Lack of energy	1.78	1.407
Bladder	Lack of energy	2.17	1.457
Stomach	Lack of energy	1.73	0.505
Liver	Constipation	2.23	1.666
Kidney	Difficulty sleeping	1.85	1.337
Pancreas	Lack of energy	2.04	1.163
Lung	Lack of energy	1.59	1.197
Breast	Lack of energy	1.89	1.118
Gynecological	Worrying	2.19	1.113
Prostate	Pain	2.16	0.496
Testicle	Worrying	2.93	1.508
Hematological	Lack of energy	1.34	0.913
Head and neck	Dry mouth	2.06	1.488
Esophagus	Pain	2.93	0.189
Melanoma	Difficulty sleeping	2.83	0.424
Others	Lack of energy	1.86	1.309

**Table 3:** Symptom descriptive of higher and lower frequency, severity and distress.

	Frequency (N (%))		Severity (N (%))		Distress (N (%))	
SYMPTOM	Rarely	Almost Constantly	Slight	Very Severe	Not at All	Very Much
Difficulty concentrating	6 (8.2)	3 (4.1)	37 (50.7)	0 (0.0)	13 (17.8)	0 (0.0)
Pain	11 (8.9)	10 (8.1)	33 (26.6)	5 (4.0)	2 (1.6)	5 (4.0)
Lack of energy	10 (5.3)	31 (16.5)	46 (24.5)	8 (4.2)	11 (5.8)	7 (3.7)
Cough	11 (18.0)	5 (8.2)	28 (45.9)	2 (3.3)	15 (24.6)	2 (3.3)
Feeling nervous	21 (15.9)	7 (5.3)	57 (43.2)	4 (3.0)	17 (12.9)	3 (2.3)
Dry mouth	14 (9.4)	19 (12.7)	56 (37.6)	13 (8.7)	23 (15.4)	8 (5.4)
Nausea	10 (15.0)	2 (3.0)	22 (32.8)	1 (1.5)	6 (8.9)	1 (1.5)
Feeling drowsy	13 (16.9)	6 (7.8)	27 (35.1)	3 (3.9)	25 (32.5)	0 (0.0)
Numbness/tingling in hands/feet	12 (9.7)	29 (23.4)	49 (39.5)	10 (8.1)	23 (18.5)	7 (5.6)
Difficulty sleeping	15 (11.8)	26 (20.5)	35 (27.6)	16 (12.6)	10 (7.9)	12 (9.4)
Feeling bloated	8 (11.8)	12 (17.6)	21 (30.9)	5 (7.4)	10 (14.7)	5 (7.3)
Problems with urination	4 (17.4)	4 (17.4)	6 (26.1)	3 (13.0)	3 (13.0)	4 (17.4)
Vomiting	8 (25.0)	0 (0.0)	14 (43.7)	0 (0.0)	5 (15.6)	0 (0.0)
Shortness of breath	13 (22.0)	6 (10.2)	23 (39.0)	3 (5.1)	4 (6.8)	5 (8.5)
Diarrhea	9 (15.5)	7 (12.0)	22 (37.9)	5 (8.6)	13 (22.4)	7 (12.1)
Feeling sad	18 (17.6)	9 (8.8)	40 (39.2)	7 (6.9)	13 (12.7)	9 (8.8)
Sweats	10 (15.6)	6 (9.4)	24 (37.5)	5 (7.8)	10 (15.6)	6 (9.4)
Worrying	22 (13.4)	18 (11.0)	54 (32.9)	13 (7.9)	15 (9.1)	14 (8.6)
Problems with sexual interest or activity	8 (8.1)	38 (38.4)	22 (22.2)	28 (28.3)	22 (22.2)	14 (14.1)
Itching	6 (11.3)	8 (15.1)	20 (37.7)	4 (7.5)	9 (17.0)	5 (9.4)
Lack of appetite	6 (7.3)	9 (11.0)	14 (17.1)	4 (4.9)	12 (14.6)	3 (3.7)
Dizziness	10 (27.0)	1 (2.7)	15 (40.5)	0 (0.0)	3 (8.1)	2 (5.4)
Difficulty swallowing	7 (14.9)	7 (14.9)	17 (36.2)	7 (14.9)	2 (4.2)	7 (14.9)
Feeling irritable	17 (16.2)	3 (2.9)	37 (35.2)	3 (2.9)	10 (9.5)	3 (2.9)
Mouth sores			27 (47.4)	4 (7.0)	6 (10.5)	5 (8.8)
Changes in the way food tasted			28 (23.0)	22 (18.0)	10 (8.2)	14 (11.5)
Weight loss			20 (36.4)	0 (0.0)	33 (60.0)	0 (0.0)
Hair loss			23 (23.0)	38 (38.0)	26 (26.0)	27 (27.0)
Constipation			29 (24.4)	12 (10.1)	13 (10.9)	9 (7.6)
Swelling of arms or legs			20 (37.7)	6 (11.3)	10 (18.9)	4 (7.5)
“I don’t look like myself”			13 (33.3)	5 (12.8)	1 (2.6)	8 (20.5)
Changes in skin			25 (36.8)	3 (4.4)	20 (29.4)	5 (7.3)
Number (percentage)						

## Data Availability

The datasets generated and/or analyzed during the current study are available from the corresponding author on reasonable request.
